# Target-Based Small Molecule Drug Discovery for Colorectal Cancer: A Review of Molecular Pathways and In Silico Studies

**DOI:** 10.3390/biom12070878

**Published:** 2022-06-23

**Authors:** Said Moshawih, Ai Fern Lim, Chrismawan Ardianto, Khang Wen Goh, Nurolaini Kifli, Hui Poh Goh, Qais Jarrar, Long Chiau Ming

**Affiliations:** 1 PAP Rashidah Sa’adatul Bolkiah Institute of Health Sciences, Universiti Brunei Darussalam, Gadong BE 1410, Brunei; saeedmomo@hotmail.com (S.M.); aifern.lim19@gmail.com (A.F.L.); nurolaini.kifli@ubd.edu.bn (N.K.); pohhui.goh@ubd.edu.bn (H.P.G.); 2 Department of Pharmacy Practice, Faculty of Pharmacy, Universitas Airlangga, Surabaya 60115, Indonesia; 3 Faculty of Data Science and Information Technology, INTI International University, Nilai 71800, Malaysia; 4 Department of Applied Pharmaceutical Sciences and Clinical Pharmacy, Faculty of Pharmacy, Isra University, Amman 11622, Jordan; Qais.jarrar@iu.edu.jo

**Keywords:** protein targets, cheminformatics, drug discovery, kinases, chemotherapy

## Abstract

Colorectal cancer is one of the most prevalent cancer types. Although there have been breakthroughs in its treatments, a better understanding of the molecular mechanisms and genetic involvement in colorectal cancer will have a substantial role in producing novel and targeted treatments with better safety profiles. In this review, the main molecular pathways and driver genes that are responsible for initiating and propagating the cascade of signaling molecules reaching carcinoma and the aggressive metastatic stages of colorectal cancer were presented. Protein kinases involved in colorectal cancer, as much as other cancers, have seen much focus and committed efforts due to their crucial role in subsidizing, inhibiting, or changing the disease course. Moreover, notable improvements in colorectal cancer treatments with in silico studies and the enhanced selectivity on specific macromolecular targets were discussed. Besides, the selective multi-target agents have been made easier by employing in silico methods in molecular de novo synthesis or target identification and drug repurposing.

## 1. Introduction

Cancer does not develop from a single gene defect in a similar way to how it occurs in other diseases such as cystic fibrosis or muscular dystrophy. Instead, cancer becomes invasive in the event that there are multiple cancer gene mutations where the safeguarding mechanisms could not protect the normal and healthy mammalian cells from their lethal effects. As a result, it is better to think of cancer genes that have been altered as contributing to, rather than causing, cancer [1]. The development of colorectal cancer involves a multiple step process incited by a distinctive genomic instability which encourages the cancerous cells to multiply, as well as increases the chances of cell survival.

Colorectal cancer has three recognized primary molecular groupings in terms of molecular genetics. The most prevalent one is the “chromosomal instable” group, which is defined by an accumulation of mutations in certain oncogenes and tumor suppressor genes. Chromosomal instability is the most common type of genomic instability in CRC. It is characterized by various changes in chromosomal copy number and structure. The normal activities of certain tumor-suppressor genes, such as APC, P53, and *SMAD4*, can be altered via a mechanism triggered by chromosomal instability which is responsible for the physical loss of a wild-type copy of these tumor suppressor genes. The second group is the CpG Island Methylation phenotype (CIMP), which is defined by DNA hypermethylation [2], as additional genes were discovered to be influenced by the process, revealing that some groupings of genes had consistently elevated methylation in particular tumors. This was proved statistically by demonstrating that the methylation of two distinct genes in a specific tumor type was associated in cases such as colorectal cancer [3].

The third group is the “microsatellite instable” (MSI) colorectal cancer thatis caused by DNA mismatch repair gene failure, resulting in genetic hypermutability. High MSI was found in 75% of this group, which is often linked with hypermethylation and *MLH1* gene silence, whereas the remaining 25% had mutations in the mismatch-repair and polymerase (POLE) genes [4]. Generally, genomic instability can cause aggregation of mutations in genes that are responsible for normal cell regulation and growth, such as proto-oncogenes and tumor suppressor genes [5]. It can also derange the normal cell repair system, induce epigenetic changes in DNA, and produce non-functional proteins that could threaten the healthy cells. Notably, the significant types of genomic instability involved in the development of colorectal cancer are chromosomal instability but microsatellite stable and microsatellite instability (MSI) [6]. Markedly, MSI is often associated with the CpG island methylator phenotype and hypermutation, which is essentially found in the right colon [7]. Furthermore, parallel investigations revealed that the mismatch repair gene *MLH1* was hypermethylated and silenced in these MSI-positive tumors. The fact that inhibiting methylation repaired the mismatch repair deficit in colon cancer cell lines supported the hypothesis that hypermethylation causes MSI through *MLH1* silencing [3]. MSI affects the size of the mononucleotide or dinucleotide repeats, which are also known as microsatellites, existing all over the genome. It occurs when the strand slippage within the repetitive DNA sequence element failed to be repaired. Such instability resulting from the loss of mismatch-repair function of proteins in DNA can further contribute to the inactivation of the tumor suppression pathway [6].

A cancerous tumor can be characterized by low frequency of somatic mutations such as single nucleotide variants (SNVs), copy number aberrations (CNAs), structural variations, and indels. As indicated by the name, SNVs are aroused by a single nucleotide variant that occurred in one particular genetic position, while CNAs are the amplifications or deletions of copies of a DNA region at a larger scale. However, structural variation is used to describe an area of DNA that is 1 kb or bigger in size and can include inversions, balanced translocations, and genomic imbalances, which are also known as copy number variations. Insertions and deletions, called indels, are changes to the DNA sequence that result in the addition or deletion of one or more nucleotides [8]. Only a small percentage of all somatic changes, known as driver mutations, offer a selective advantage to cancer cells, whereas the vast majority of somatic mutations are passenger mutations that do not contribute to the illness [9]. Inter-tumor heterogeneity, where cancer genomes do not share a similar set of somatic mutations and most of the different metastatic tumors bear a different kind of mutation in the same patient, is the most remarkable trait of the cancer mutational landscape [10]. Besides, in less than 5% of all patients with a specific cancer type, a small number of gene mutations are found in a large portion of tumors and mostly are affected by SNVs or CNAs [11]. Inter-tumor heterogeneity impedes efforts to discover driver genes with driver mutations by recognizing commonly mutated genes that are mutated in a statistically high proportion of patients [12]. The nature of the driver mutations in targeting normal functional genes, groups of interacting proteins, as well as signaling and molecular pathways, is one of the causes of inter-tumor heterogeneity [13].

In silico techniques have long been considered crucial in the efforts of predicting inhibitors, new targets, and diagnostic tools for CRC treatment plans. Exploring binding pockets, residue interactions, and different virtual screening methods are approaches, among others, that were utilized to target CRC [14]. Gene-mutated CRC was targeted by topological in-silico simulations to predict the best treatment combinations that can be successful in clinically advanced conditions [15]. Furthermore, other tactics, such as the simulations that predict the interplay between tumor microenvironment components, could enhance or reduce immunotherapy success or failure [16], and the gut-on-chip model that delineates the molecular mechanism of symbiotic effects on CRC genes’ expression [17] are examples of significant accomplishments in this field. The use of computational methods has also proved a distinguished efficacy by analyzing cell surface proteins overexpression in predicting disease progression, diagnosis, and drug resistance in CRC [18]. MicroRNA was employed as a biomarker for CRC through its attachment to the predicted target gene. The molecular pathways and functional analysis of this non-coding RNA with its target macromolecules can predict CRC pathogenesis [19]. In this review, we summarized the molecular pathways involved in colorectal cancer and the main driver genes that have the greatest triggering impacts. We also discussed the main tumor suppressor genes that can be inactivated, such as APC, TP53, and TGF-β, mainly the growth factor pathways VEGFR and EGFR, and the microsatellite instability mechanism involving genes. In each pathway, an overview of some landmark virtual screening studies that involves finding hits and/or optimizing lead compounds for each individual protein target were provided.

## 2. Driver Genes in CRC

**Figure 1:**
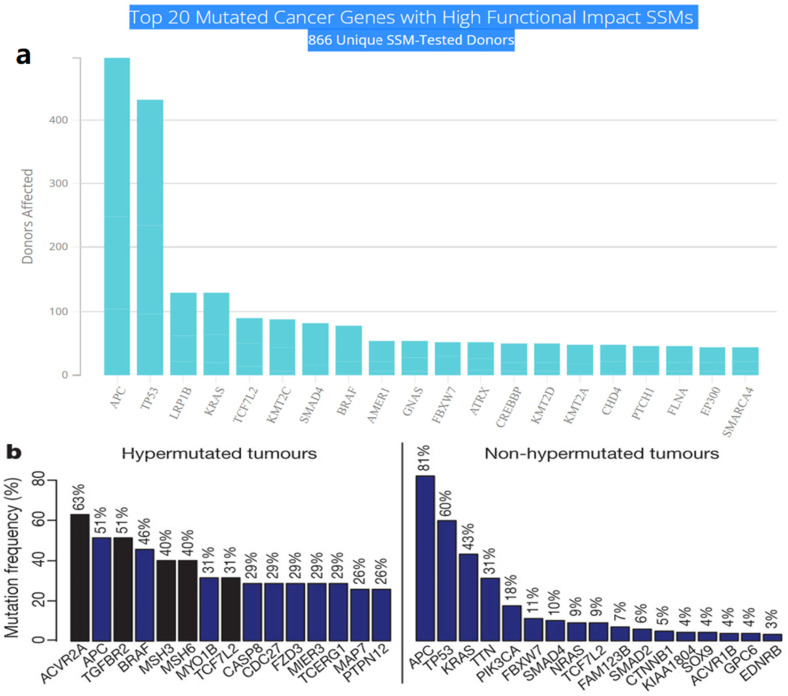
(**a**) The top 20 mutated genes with high functional impact involved in colorectal cancer extracted from the ICGC Data Portal in three projects: Colon Adenocarcinoma—TCGA, US, Adenocarcinoma, non-Western (China), Rectum Adenocarcinoma—TCGA, US. https://dcc.icgc.org/ (accessed on 15 December 2021) (**b**) Significantly mutated genes in hypermutated and non-hypermutated tumors adopted from The Cancer Genome Atlas Network [7].

Multistep tumorigenesis develops through the gradual collection and alterations of driver genes in colorectal cancer. Less than 1% of human genes can potentially turn into cancerous driver genes which are actively capable of controlling cell survival and fate, as well as affecting normal genome stability [10,20]. For a mature cell to become cancerous, it has to undergo phases of breakthrough, expansion, and invasion within 20 to 30 years, involving at least 2 to 3 driver gene mutations. It begins with the first driver mutation which minimally benefits the cell to survive and turns into a proliferating hyperplastic lesion. This could increase the risk of acquiring the second driver gene mutation and further leads to the third driver gene mutation as the cell gained autonomy and immortality, as well as the ability to self-renew. In the case when a third driver gene is involved, the tumor cell is upgraded to become invasive and metastatic. At this point, the malignant cells disseminate without the assistance of other driver mutations [21]. The International Cancer Genome Consortium (ICGC) platform shows the top 20 mutated genes in CRC such as APC, TP53, LRP1B, KRAS, and BRAF, which are significantly impacted by single somatic mutations that also have high functional impact as shown in Figure 1a. ICGC is a global platform that has compiled data on 670,946 unique somatic mutations and molecular profiles from 866 donors for CRC patients. These collected data are grouped into three CRC-related projects, namely, colon adenocarcinoma—TGCA, USA (COAD-US), non-Western colorectal cancer—China (COCA-CN), and rectum adenocarcinoma—USA (READ-US). In the same context, the Cancer Genome Atlas project profiled genomic changes in three cancer types; glioblastoma and ovarian carcinoma, in addition to colon and rectal cancer, among 20 different cancer types with a comprehensive molecular characterization for each one of them [7]. In this project, 276 samples were analyzed for a genome-scale investigation of promoter methylation, exome sequence, DNA copy number, and messenger and microRNA expression. Frequent mutations were revealed in ARID1A, SOX9, and FAM123B, in addition to the expected APC, TP53, *SMAD4*, PIK3CA, and KRAS mutations as shown in Figure 1b. Furthermore, amplifications in ERBB2 and the “newly-discovered” IGF2 that might be drug-targeted were also identified in the same project, are two examples of recurrent copy-number alterations.

**Figure 2:**
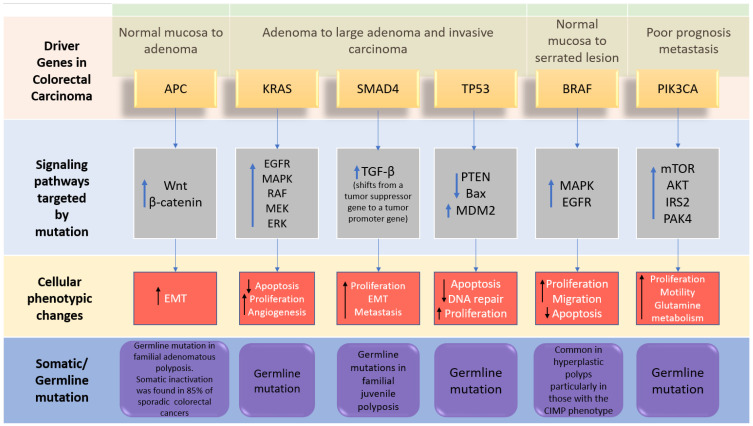
The driver genes and signaling pathways involved across the CRC adenoma–carcinoma sequence from the transition of normal epithelium through to the metastasis stage in colorectal cancer (adopted from [6]). IRS2; insulin receptor substrate 2, MDM2; Mouse double minute 2 homolog, mTOR; Mammalian target of rapamycin. PAK4; p21 (RAC1) activated kinase 4, EMT; epithelial–mesenchymal transition.

The genome-wide investigations strongly confirm the links between commonly altered driver genes and human colorectal cancer (Figure 2). Tumorigenesis is generated in the presence of mutant driver genes such as APC, KRAS, *SMAD4*, TP53, PIK3A, ARID1A, and SOX9, in intestinal epithelial cells using organoid culture systems [7,22]. In addition to the previously stated genes, other changed genes identified to be implicated in colorectal cancer carcinogenesis include FBXW7, BRAF, TCF7L2, PIK3CA, GNAS, CBX4, ADAMTS18, TAF1L, CSMD3, ITGB4, LRP1B, and SYNE1 [23]. APC, KRAS, BRAF, PIK3CA, *SMAD4*, and TP53 are the six CRC driver genes, with APC, KRAS, PIK3CA, and p53 being the most often altered. Mutations in APC, KRAS, and BRAF occur early in the transition phase from normal epithelium to adenoma, whereas PIK3CA mutation and loss of *SMAD4* and P53 (due to mutations or epigenetic silencing) occur late, allowing tumor cells to invade surrounding tissues and metastasize, transforming the adenoma into a carcinoma. Mutations in APC, TP53, and KRAS, as well as, to a lesser extent, *SMAD4*, are related to metastatic conditions while being highly associated with MSI [24]. The APC (adenomatous polyposis coli) gene is thought to be the gatekeeper gene for CRC, with mutations reported in 83% of all cases [25]. KRAS contributes significantly to carcinogenesis by activating the RAF–MAPK and PI3K pathways. TGF-β signaling, on the other hand, promotes epithelial cell differentiation, acting as a tumor suppressor in colorectal cancer. Furthermore, FBXW7 is a component of the ubiquitin ligase complex, which eliminates proto-oncogene products by degradation, acting as a tumor suppressor, and Fbxw7 disruption promotes intestinal carcinogenesis. According to recent findings, mutant p53 affects gene expression globally via a gain-of-function mechanism, which promotes cancer [22]. APC mutations frequently occur concomitantly with KRAS or TP53 mutations, or both. This triad predicts poor prognosis, whereas BRAF, ITGB4, CBX4, CSMD3, SYNE1, FBXW7, and TAF1L are substantially linked to MSI but not to metastatic illness [20].

## 3. Inactivation of Tumor-Suppressor Genes

### 3.1. Adenomatous Polyposis Coli (APC)

**Figure 3:**
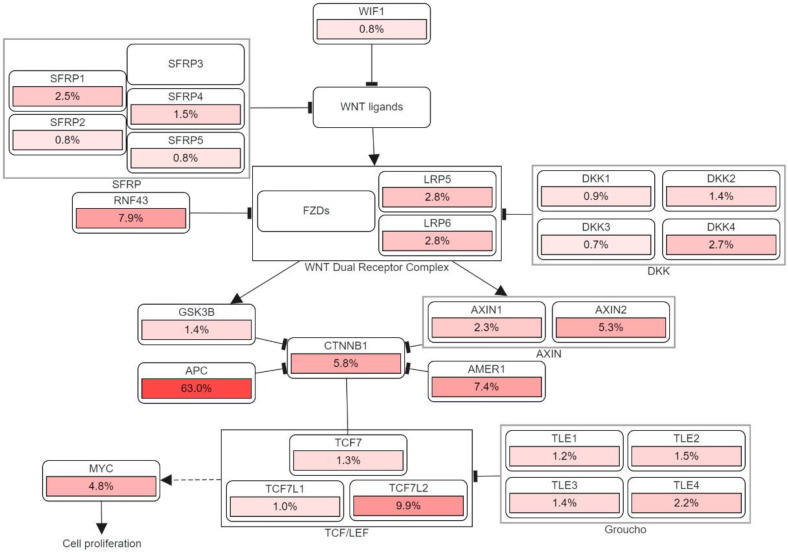
The genetic pathways and frequencies of mutations collected from 13 studies and 4535 samples in the cBioportal platform that results in deregulation in Wnt signaling pathway, leading to the cell phenotypic modification. The dotted arrow illustrates induction. CTNNB1: Catenin Beta 1, TCF7: Transcription Factor 7, DKK: Dickkopf WNT Signaling Pathway Inhibitor, LRP: LDL Receptor Related Protein, SFRP: Secreted Frizzled Related Protein. The percentage under each gene represents the percent of mutated/altered samples related to the profiled ones in those studies [30,31,32,33,34,35,36,37,38].

Apart from generating familial adenomatous polyposis (FAP), mutations in both alleles of the APC gene have a rate-limiting role in most sporadic CRC. The cascade of molecular events induced by the loss of APC function can subsequently contribute to the malignancy of the large bowel [26]. One of the crucial intracellular components, β-catenin, which is also the binding partner of APC, is found to be involved in the Wingless/Wnt signal transduction pathway. Wnt signaling pathway, which is promoted by the mutation of gene encoding the APC protein, initiates genomic colorectal carcinogenesis. Normally, the unoccupied, phosphorylated β-catenin is attached to the destruction complex in healthy cells without being stimulated by the extracellular Wnt signal. The destruction complex consists of the scaffolding protein axin, as well as other components such as APC, conductin, and glycogen synthase kinase 3-β (GSK3β). If not attached to that complex, the nuclear localization of β-catenin proteins will create a transcription factor favoring the cellular activation of oncogenic activities. Therefore, as the APC protein complex loses its function due to its encoding gene mutation, Wnt signaling pathway is activated with increasing oncogenic β-catenin protein nuclear localization. Somatic mutations and deletion of APC encoding gene are discovered in most sporadic colorectal adenomas and carcinomas, while germ-line mutations were found in familial adenomatous polyposis [6,27]. Figure 3 illustrates the detailed pathway.

CyclinD1 and MYC are the first two discovered downstream targets in Wnt signaling pathway responsible for tumor formation due to their capabilities in cell apoptosis, proliferation, and controlling or disrupting cell-cycle progression. Direct and indirect Myc activation via the Wnt/β-catenin pathway have distinct carcinogenic effects in the intestinal epithelium [28]. On the other hand, β-catenin overexpression in the cytoplasm, may accelerate malignant transformation in colorectal tumors by stimulating cyclin D1 expression [29]. Other Wnt target genes, including matrilysin, CD44, and the urokinase-type plasminogen activator receptor, appear to be more involved in tumor promotion than in tumor initiation [26].

### 3.2. TP53 Inactivation Pathway

Generally, the most frequent type of gene alterations that occur in human cancers are the p53 gene mutations. The transcriptional activity of the p53 protein is inactivated in most colorectal cancers by a missense mutation of the first allele and a 17p chromosomal deletion that extinguishes the second allele. The functional domains of TP53 are: transactivation domain (TAD), core domain that identifies specific DNA sequences, tetramerization domain, and the C-terminal domain that is responsible for the regulation of p53 activity [39]. As both p53 alleles are eliminated, tumor suppression activities in its pathway were shut down and the existing large adenomas become more invasive. The activity of p53 pathway can also be suppressed by the mutation in gene encoding BAX, which normally induces cell apoptosis, in colorectal cancers with mismatch-repair defects [40]. P53 protein is a stress-inducible transcription factor, acting as a functional regulator in a variety of downstream genes in multiple cell-signaling processes. In order to control the level of p53 from being excessive in normal cells, the negative regulator of p53 i.e., MDM2 will be upregulated to degrade p53 by regulating the ubiquination of p53. An abnormal amount of p53 can lead to cell apoptosis, cell cycle arrest or senescence triggered by DNA damage, hypoxia, and oncogene activation, as well as other cellular stresses [41].

Two pathways are triggered simultaneously upon the activation of p53, namely, the intrinsic mitochondrial and the extrinsic death-receptor-induced apoptotic pathways. Down along the intrinsic pathway, the pro-apoptotic B-cell lymphoma-2 (Ccl-2) family proteins (i.e., BAX, Noxa and PUMA) are induced while the pro-survival Bcl-2 are downregulated instead. As the result of the permeabilization of its outer membrane, the substance cytochrome c, which is released from the mitochondria, binds to Apaf-1 and forms a complex. The complex then activates initiator caspase-9, followed by executioner capase−3, −6, and −7 [42]. In the extrinsic pathway, the expressions of death receptors (DFs) Fas (CD95/APO-1), DR5 (TRAIL-R2), and PIDD (p53-induced protein with death domain) are upregulated as p53 is activated [43]. Additionally, a co-transcription factor named AFT3 assists p53 in maximizing the expression of DR5, which is a trans-membrane tumor necrosis factor (TNF), in CRC induced by DNA damage. DR5 consists of a death domain which binds to the tumor necrosis factor-related apoptosis-inducing ligand (TRIAL) and activates the extrinsic apoptotic pathway that triggers cell death [44].

A variety of small compounds have been designed to target and stabilize certain mutant versions of p53, restoring wild-type (WT)-like transcriptional activity and causing mutant tumor cells to undergo cell cycle arrest or apoptosis. The nine most common mutations of p53 protein (R175H, R248Q, R273H, R248W, R273C, R282W, G245S, R249S, Y220C) account for around 30% of all its cancer-driving mutations [45]. PRIMA-1 and its methyl analog APR-246 are potential small molecules that interact with the DNA binding domain of mutant p53, encouraging correct folding/function and, as a result, increase the production of pro-apoptotic genes Puma, Noxa, and Bax in p53 mutant cells [46]. The Y220C mutation is the ninth most common p53 missense mutation, that is linked to more than 100,000 new cancer cases each year. The Y220C pocket’s hydrophobic and “druggable” characteristics make it a good candidate to be targeted by small-molecule stabilizers. The mutation-induced crevice is far away from the p53 surfaces involved in DNA recognition or protein–protein interactions, allowing for creation of tailored chemical agents that stabilize the DNA binding domain without interfering with its natural substrate binding [45]. Several powerful lead compound families that bind Y220C pockets have been identified in recent years using fragment-based and in silico screening approaches. PK9328 is a carbazole derivative that was identified by computational screening techniques fit in the p53-Y220C binding pocket with a low micromolar affinity and has a significantly decreased cell viability in various Y220C cancer cell lines [47]. Moreover, the pyrazole derivative PK7088 restored p53-Y220C transactivation and downstream upregulation of p21 and Noxa expression, correlated with cell cycle arrest and apoptosis [48].

### 3.3. TGF-β Tumor Suppressor Pathway

Because it affects cell proliferation, differentiation, apoptosis, and homeostasis, TGF-β signaling is critical in the context of inflammation and cancer. TGF signaling suppresses epithelial growth in normal tissues but promotes tumor cell proliferation in malignant tissues. This phenomenon is called the TGF-β paradox, and instead of its typical nature of inhibiting the epithelial growth in normal tissues, the activated signaling pathway stimulates tumor progression in cancerous cells [49]. Tumor cells’ release of TGF-β also reduces the immune response to the tumor, allowing it to develop further [50]. Two serine/threonine protein kinases (Type I and Type II receptors) and a series of downstream substrates (SMADs) are involved in TGF-β signaling. Type 2 receptors work as activators to phosphorylate type I receptors, and type 1 operate as propagators to carry the signal downstream to cytoplasmic proteins [51]. Bone morphogenetic protein (BMP) type 1 receptors phosphorylate SMAD1/5/8 after ligand binding, whereas TGF- type I and activin type 1 receptors phosphorylate SMAD2/3. These sets of SMAD proteins are known as receptor-regulated SMAD (R-SMAD). Trimerization with a common-mediator *SMAD4* and two R-SMAD molecules, which is facilitated by the phosphorylation of two C-terminal serine R-SMAD residues, leads to its translocation into the nucleus to bind to the DNA binding site [52]. The other non-canonical, SMAD-independent pathways that can be transduced by the TGF-β superfamily ligands include phosphoinositide 3-kinase (PI3K)/Akt, Rho/Rho-associated protein kinase (ROCK) pathways, as well as multiple types of mitogen-activated protein kinase (MAPK) [53].

TGFBR2 mutations are frequently found in MSI-H CRC (colorectal cancer with microsatellite instability-high frequency). Mismatch repair genes are silently expressed in MSI-H CRC cells due to germline mutations in genes such as MutL homolog 1 (*MLH1*), MutS homolog 2 (*MSH2*), *MSH6*, and Postmeiotic segregation increased 2 (*PMS2*), or *MLH1* promoter hypermethylation. The genes that are affected by the germline mutations are usually MutL homolog 1 (*MLH1*), MutS homolog 2 (*MSH2*), MutS homolog 6 (*MSH6*), Postmeiotic segregation increased 2 (*PMS2*) or *MLH1* promoter hypermethylation. TGFBR2 mutations, which are often discovered in MSI-H CRC, have the ability to convert normal epithelial cells into malignant ones in the colon [54]. Therefore, the malignant phenotype of the affected CRC cells will arise via Hippo, MAPK, and Wnt-β-catenin signaling pathways [55]. The second type of TGF-β Signaling in CRC is the mutation and deletion of the suppressor gene *SMAD4* as a key transcription factor in this pathway. Many genes in the 18q21 chromosomal region are frequently affected by the loss of heterozygosity including *SMAD2* and *SMAD4* may contribute to forming microsatellite-stable CRC. Because it is a transcription factor for TGF-β signaling, the loss of tumor suppressor gene *SMAD4* impairs canonical TGF-β signaling [7]. The non-canonical TGF-signaling route is the third signaling pathway. Although *SMAD4* deletion inhibits canonical TGF-β signaling, it modifies BMP signaling via a non-canonical route to enhance CRC metastasis via activation of the Rho/ROCK pathway, resulting in EMT, migration, and invasion. *SMAD4* deficiency also activates alternate MEK/ERK pathways, promoting cell death, migration, and invasion [56].

The three above-mentioned inactivation of tumor suppressor genes pathways have witnessed many attempts to develop inhibitors against a certain molecular signaling that was inhibited by the APC, TGF-β, and other genes. In Table 1, we collected a number of representing in-silico studies by computer aided drug discovery and high throughput virtual screening to show the targets that were used and the results of these studies. Due to fundamental roles played by TGF-β suppressor gene, its downstream pathways, and the diverse mutations on its main pathway components, many computational approaches were considered to identify potential small molecules to restore is original function. Nicklas et al. [57] established a computer modeling-based technique capable of statistically analyzing the signaling cascade in order to identify possible treatment targets. They investigated a model that incorporated the exact dynamics of the system, mutations that impact system parameters, and a collection of potentially targetable pathway components, such as the suppression of protein association or production. Interestingly, they also found a collection of mutations that significantly change the signaling dynamics for each cell line, as well as a number of molecular interventions that may be employed to effectively target the effects of these mutations, based on the findings of the molecular intervention optimization method. In a different manner, other in silico studies were established to study the negative regulation on the TGF-β/Smad signaling system on different time scales [58]. This also includes a set of computer models that illustrate the individual and combined impacts of R-Smad negative regulation. Comparisons of models and data indicated that negative regulation occurs at several temporal scales. It has been revealed that a model would need to include at least one fast-mode and one slow-mode effect in order to describe the phospho-R-Smad dynamics in both short- and long-exposure studies. A second important discovery in the aforementioned study was a unique negative feedback effect, which has been verified experimentally, in which the phosphatase PPM1A is increased following TGF- β stimulation. Another addition provided by the same study is an explanation for an earlier debate over proteasomal degradation of phospho-R-Smad. Nevertheless, studies that inhibited proteasomal degradation reported either substantial or no impact on phospho-R-Smad levels. Both of these seemingly contradicting tendencies were mathematically compatible with the mentioned model, and the gap may be explained by varied TGF- β exposure durations.

## 4. Growth Factor Pathways

The main growth factor pathways include vascular endothelial growth factor receptor-2 (VEGFR-2) and epidermal growth factor receptor (EGFR), as well as other protein kinases.

### 4.1. Vascular Endothelial Growth Factor Receptor-2 (VEGFR-2)

**Figure 4:**
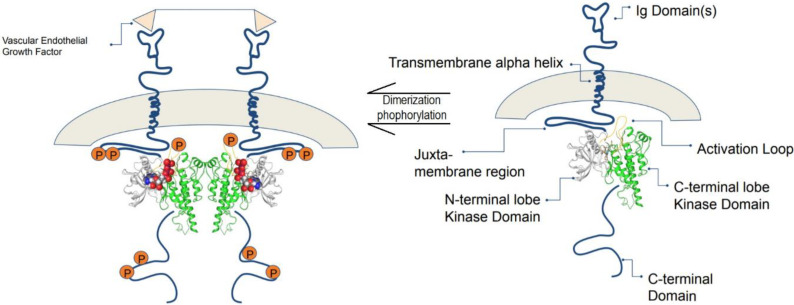
The composition of VEGFR consists of seven immunoglobin-like motifs. VEGF binds to the extracellular domain, and VEGFRs dimerize, leading to a conformational change that is transmitted across the membrane, which leads to activation. Adapted from Schrodinger tutorials [86].

A majority of central cellular activities are carried out by a total of 518 protein kinases present in the human genome which account for about 2% of all human genes [80]. The protein data bank (PDB) has collected 185 unique structures of human protein kinase domain as well as 197 kinases of other species [81]. VEGF protein kinases are greatly involved in many vascular physiologies, such as the development of blood vessels, formation of lymphatic vessels, and homeostasis. Among the VEGF family, VEGF-A, which is also known as vascular permeability factor, is significant for angiogenesis synchronization and vasculogenesis during embryonic development. In addition, VEGF-A plays a substantial role in repairing the function of damaged tissues [82]. However, it could aggravate cancer in the event of an “angiogenic switch”, which occurs due to the imbalance in anti- and pro-angiogenic activities induced by the recruitment of inflammatory cells into the tumor cells [83]. VEGF-A mediates its biological response through VEGFR2, therefore, it is believed that the protein tyrosine kinase VEGFR2 is a potential target for anti-cancer therapy, as it acts as a medium for VEGF-A to exert its biological activities [84]. A large number of α helical C-terminal lobes, together with smaller portions of β strands-filled N-terminal lobe, construct the catalytic protein kinase domains. In the cleft between the two lobes, an active site which is surrounded by a flexible activation loop on its circumference exists. The activation loop is made of a polypeptide which usually consists of serine, threonine, or tyrosine residues that are ready to be phosphorylated. As phosphorylation occurs, the catalytic activity in the protein kinases will increase dramatically (Figure 4) [85].

According to the pattern of conformations, the protein tyrosine kinase inhibitors are classified into 4 types: Type I, Type II, Type III, and Type IV [87]. The competitive Type I and II enzyme inhibitors, which interact with ATP-binding pocket and Mg2+ ion in the active site of the domain between N-terminal and C-terminal lobes, work in the presence of ATP. Type II inhibitors, specifically, extend to new pockets generated by flipped DFG motif next to ATP-binding pocket, and this pocket is formed by DFG motif rearrangement in the inactive conformation [88]. Type II inhibitors have an advantage over type I inhibitors in that they are selective inhibitors with greater chemical space to be exploited compared to type I inhibitors [87]. Despite the high sequence conservation throughout this huge protein family, the breakthrough drug imatinib demonstrated some years ago that the flexibility of kinase structure can permit the generation of specific kinase inhibitors. Imatinib is classified as a “type II” kinase inhibitor because it binds to both the ATP cofactor binding site and an adjacent “allosteric” site that is only available when the kinase adopts a catalytically inactive conformation in which the “Asp-Phe-Gly (DFG)” motif at the N terminus of the activation loop is flipped “out” [89]. Type I inhibitors, such as dasatinib, bind at the ATP site but not the allosteric pocket, hence they are not dependent on certain kinase conformations for binding. Hari et al. [85] address this matter, arguing that underlying disparities in kinase capacity to adopt the DFG-out conformation might contribute to type II inhibitor selectivity.

**Figure 5:**
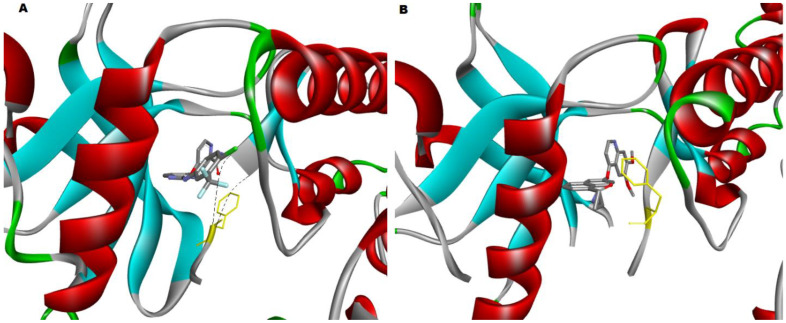
(**A**) The crystal structure of the VEGFR2 kinase domain in complex with a benzimidazole inhibitor (2QU5) has the phenylalanine (highlighted in yellow) of the DFG motif facing much closer to the surface of the active site; therefore, it is in the inactive DFG-out state, and (**B**) The crystal structure of the VEGFR2 kinase domain in complex with a naphthamide inhibitor (3B8R), showing that the DFG motif has the phenylalanine (highlighted in yellow) facing in towards the center of the pocket between the N-lobe and C-lobe; therefore, it is in the active DFG-in state. The two PDB-derived structures were visualized by Discovery Studio v21.1.

Magnesium ion-ATP is positioned in a deep cleft between the N- and C-terminal lobes of the highly conserved kinase domain. The bulk of small-molecule kinase inhibitors produced to date target the ATP binding site, with the kinase assuming a conformation that is almost comparable to that of the ATP binding site (the active conformation). The discovery of a second family of kinase inhibitors, whose members preferentially bind to an inactive conformation of the kinase, blocking activation, has been made possible through medicinal chemistry [90]. Type II inhibitors exploit the ATP binding cleft and a nearby hydrophobic pocket generated by the activation loop’s “out” conformation (which contains the conserved DFG motif). Type I inhibitors attach to the ATP binding site by forming hydrogen bonds with the kinase “hinge” residues and by hydrophobic contacts in and around the adenine ring of ATP [91]. Type II inhibitors primarily target the ATP binding site, but they also take advantage of unique hydrogen bonding and hydrophobic interactions enabled by the activation loop’s DFG residues being folded away from the ATP phosphate transfer conformation, as shown in Figure 5 [87].

Many virtual screening campaigns were established to design potent inhibitors for VEFGR2. Virtual screening uses computer models to assess a specific biological activity of compounds in order to filter existing databases or virtual libraries for the purpose of identifying molecules that have a specific activity against the target of interest. Pharmacophoric, docking, and shape similarity screening studies are carried out in a different setting in order to optimize leads suitable for VEGF receptor-2. Table 2 summarizes the known VEGFR2 inhibitors, their PDB ID, and the effect of those inhibitors against other receptor tyrosine kinases. Additionally, Table 3 shows some in silico campaigns to find hits that can be possible inhibitors against VEGFR2. Since natural products offer immense promise in drug development as the largest source of novel molecules with active biological activities, natural products no doubt continue to be a key part of drug discovery, as they are generally perceived as less toxic. On the contrary, synthetic small molecules and monoclonal antibodies have exhibited a more severe adverse drug reaction profile. In the treatment of cancers by targeting VEGFR-2, bevacizumab, for example, is likely to produce significant ophthalmic inflammation [92], whereas sunitinib can cause multiple adverse drug reactions, including thrombopenia and hypertension [93]. Based on this, many virtual screening and computer aided drug discovery campaigns were initiated to find VEGFR-2 inhibitors based on natural products and natural products library of compounds. Sharma et al. [94] established ligand-based pharmacophore models from the most potent VEGFR-2 inhibitors, then screened a library of 62,082 natural compounds from InterBioscreen natural compound database. The yielded results were passed through many filters to guarantee good binding affinities, biological activity prediction, drug-likeness study, ADMET prediction, and molecular dynamic simulations. Others studied the FDA-approved anti-malarial artemisinin derivatives to be repurposed against VEGFR-2 and other cancer targets [95]. Artemisinin, artenimol, artemether, artemotil, and artesunate were found to interact more potently with CDK-6 and VEGFR-2 than other receptors, in addition to other density functional theory calculations that provided good insight on the electronic and structural properties, as well as various reactivity measures. Furthermore, designing inhibitors that may interact with several cancer targets at the same time, is a promising technique; hence, blocking these three receptor tyrosine kinases (RTKs) with a single chemical component may provide an effective and safe chemotherapeutic option. The polypharmacology of the flavonol “cediodarin” against three RTKs was performed by structure-based pharmacophore mapping and virtual screening of natural products library of compounds. Good affinity results were found for cediodarin against c-MET, EGFR, and VEGFR-2 [96].

### 4.2. Epidermal Growth Factor Receptor (EGFR)

Epidermal Growth Factor Receptor (EGFR), which is also known as human EGF receptor (HER), is a 170-kilodalton transmembrane cell-surface receptor with c-erb-B proto-oncogene-encoded tyrosine kinase activity [104]. EGFR acts as a catalyst in the transfer of phosphate molecules from ATP to the active site of tyrosine kinase. The resulting signals trigger cellular activities such as anti-apoptotic, tumor cells invasion, and angiogenesis promoting reaction. Subsequently, the intracellular EGFR signaling pathway is initiated together with the activation of AKT and STAT proteins as well as MAP and SRC family kinases. Thus, it further amplifies the transcription of genes that promote cell division and increase survival rate [105]. The overexpression of EGFR protein is discovered in 25 to 75 percent of colorectal cancers with poor prognosis and a high risk of developing metastasis. [106]. Furthermore, EGFR and its ligands, epidermal growth factor (EGF), and transforming growth factor-α (TGF-α) are usually co-expressed at a high level in malignant tissue compared to those in the surrounding mucosa [107]. Generally, such a phenomenon is connected with severe disease or aggressive conditions such as advanced tumor stage cancer with major mesenteric lymph-node involvement [108].

All of the EGFR family members are designated with a greatly glycosylated extracellular region containing 11 sites for glycosylation across 620 amino acids approximately. Each transmembrane domain consists of 23 residues with a juxtamembrane regulatory domain on each side, linking down to a TK domain and C-terminal regulatory region of 232 amino acids [109]. ErbB extracellular region is made up of 4 protein domains: domains I, II, III, and IV [110]. Domains II (CR1) and IV (CR2) are rich in cysteine. Furthermore, leucine-rich domains I and III are favored as binding sites for their competent growth factor ligands. On top of that, numerous studies have shown a variety of mutated EGFR coupled with domains I and III provide a high-affinity binding site for EGF [109]. The activation of EGFR results in a downstream signaling cascade of several pathways such as the RAS-RAF-MAP kinase, phosphatidyl inositol-3-kinase (PI3K), and AKT pathway as well as the activation of other malicious oncogenes such as KRAS, BRAF, MEK, and MAPK [53]. The phosphorylation of phosphatidylinositol-2-phosphate (PIP2) to phosphatidylinositol-3-phosphate (PIP3) leads to the activation of AKT and initiation of carcinoma [111,112].

The strategy of targeting the allosteric site with potent small molecule EGFR degrader has obtained more selective cancer cell killing, disrupting aberrant signaling in mutant tumors and reducing drug resistance. EAI045 is a fourth-generation allosteric EGFR inhibitor that binds away from the ATP-binding site rather than relying on Cys 797 binding. Patel et al. [113] described compound ZINC20531199 as an allosteric inhibitor to overcome the EGFR T790M/C797S Tyrosine Kinase mutation problem using virtual based screening methods. The docked compound was also shown to be stable in the allosteric pocket of the C797S EGFR tyrosine kinase after a 10-ns molecular dynamics simulation. Another attempt was carried out to target the allosteric binding site of C797S mutant EGFR enzyme [114]. Subsequently, the discovery of a Y-shaped structure has paved the way for the development of allosteric fourth-generation EGFR inhibitors. Various enumeration libraries, such as scaffold hopping and R-group enumeration, assisted in the construction of as many novel structural compounds as is feasible. The screening of chemicals from the enumerated library yielded promising allosteric inhibitor hits. Different filters, such as Lipinski’s Rule of Five, ADMET filters, and Jargan’s Rule of Three, were used to further screen the top docking score compounds. The top potential hit was put through a molecular dynamic simulation, which validated the compound’s binding ability and potency. Top-ranked virtual hit compounds binding to the allosteric site of the EGFR enzyme can function as strong EGFR inhibitors in the treatment of non-small cell lung cancer mutations. Moreover, the binding of glucokinase activator to EGFR C797S was investigated using structure-based virtual screening, which revealed that mutant-selective allosteric inhibition might overcome EGFR resistance. EAI045 was shown to be an allosteric, non-ATP competitive inhibitor of mutant C797S EGFR with a Y-shaped structure. Glucokinase activators meet all pharmacophoric requirements, similar to EAI045, and they also occur in a Y-shaped structure, similar to the allosteric inhibitor EAI045, according to a 3D pharmacophoric search. A library of 143 glucokinase activators was tested against all forms of mutant EGFR (C797S, T790M, L858R, TMLR) and WT EGFR, yielding seventeen compounds found to be potential inhibitors for all mutant EGFR in addition to wild type EGFR [115].

### 4.3. Other Receptor and Protein Kinases in CRC

**Figure 6:**
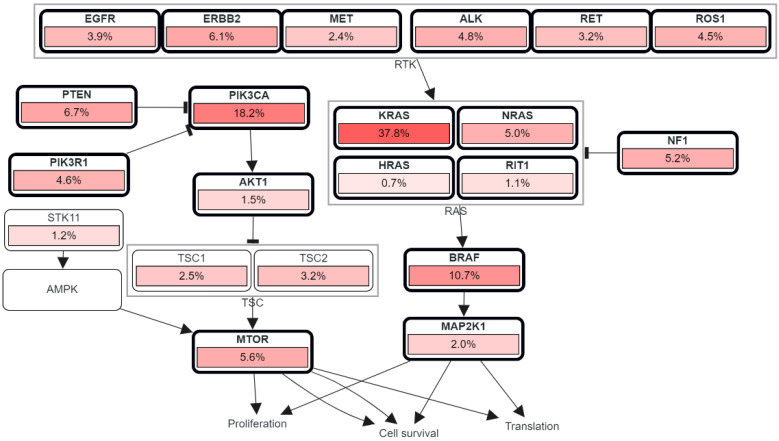
RTK, RAS, and PI3K signaling in colorectal cancer showing the genetic pathways and frequencies of mutations in 13 studies and 4535 samples in cBioportal platform that led to deregulation in this pathway reaching the cell phenotypic modification. The percentage under each gene represents the percent of mutated/altered samples relative to profiled ones in those studies [30,31,32,33,34,35,36,37,38].

The Ras-Raf-MAPK/ERK kinase and extracellular signal-regulated kinase 1 and 2 (ERK1/2) are two of the most dysregulated signaling cascades in human cancer, which are included by the MAPK pathway. In addition to the growth factors and cytokines which act via receptor tyrosine kinase signals, RAS and RAF genes mutation can also activate the RAS-RAF-MEK-ERK pathway [116]. Ras and its isotopes NRas, HRas, and Kras, in particular, bind to GDP and are inactive (‘off’ state) in normal quiescent cells, while it binds to GTP (“on” state) in response to external stimuli, which possesses an additional phosphate group. Ras binds GTP to Raf and mobilizes the inactive protein from the cytoplasm, where it recruits the Raf kinases (ARAF, BRAF, and CRAF) to the plasma membrane [117]. Ras also stimulates the serine/threonine kinase action of Raf isoforms after the Ras—Raf complex is translocated to the cell membrane. On the other hand, Raf functions as a MAPK kinase kinase (MAPKKK) when Ras is recruited, activating MEK1 and MEK2, which then catalyze the activation of the effector ERK1 and ERK2 kinases, as well as their translocation into the nucleus. Upon activation, ERK1/ERK2 phosphorylates a number of nuclear and cytoplasmic effector genes involved in a variety of physiological responses, including cell proliferation, survival, differentiation, motility, and angiogenesis [118]. Other downstream signaling pathways that Ras can activate include PI3K, p38 MAPK, and the JNK stress-activated protein kinase pathway. Furthermore, the phosphoinositide 3-kinase (PI3K) enzyme is involved in cancer cell proliferation, survival, and motility/metastasis. Phosphoinositide-dependent protein kinase-1 (PDK1), Akt, the mammalian target of rapamycin (mTOR), and the ribosomal protein S6 kinase (S6K) are all involved in PI3K signaling, which governs cell growth, proliferation, and survival. The fact that mutations in the tumor suppressor gene PTEN are common in human cancers emphasizes the relevance of PI3K/Akt/mTOR signaling in cancer [119,120], as depicted in Figure 6.

On top of that, IGF-2 has been proposed to act as an auto-/paracrine growth factor in human CRC via binding to IGF-1R. IGF-1 promotes the production of vascular endothelial growth factor (VEGF) in human colon cancer cells by inducing VEGF gene transcription. IGFs are also anti-apoptotic compounds that play a role in cell proliferation and the renewal of epithelial cell populations [121]. Among 22 known ligands of the fibroblast growth factors (FGFs) family, there are 5 highly conserved transmembrane tyrosine kinase receptors (FGFR1-5) that were identified. FGFs interact with the cell surface and its cellular matrix via heparan sulphate proteoglycans (HSPGs) stabilization [122]. A cascade of downstream signaling pathways, such as DAG-PKC and IP3-Ca2+ signaling branches via PLCγ activation, mitogen-activated protein kinase (MAPK), phosphoinositide-3-kinase (PI3K)/Akt pathways, and signal transducer and activator of transcription (STAT), are triggered upon ligand binding and dimerization of FGFRs [123]. Similar to most of the signaling pathways mentioned, FGFR pathway activation contributes to carcinogenesis with somatic abnormalities [124]. The causes of FGFR overexpression include gene alterations (i.e., point mutations and translocations) in the process of post-transcription which results in constitutive activation of receptors or diminished sensitivity in ligand binding as well as production of fusion proteins with uncontrolled cellular activities. Other than that, isoform switching and alternative splicing, which reduces FGFs specificities, can also lead to FGFR overexpression [125]. In Table 4, we summarized some of the receptor tyrosine kinases with examples for virtual screening studies for discovering new lead compounds to the respective receptor/protein.

## 5. Microsatellite Instability Pathways

### 5.1. Epigenetic Silencing of Gene Expression

In the process of DNA methylation, the enzyme DNA methylase introduces a methylated form of cytosine to the 5′-position as the fifth DNA base by modifying the cytosines within the CpG dinucleotides. In adult cells, the majority of the remaining CpG sites are methylated. A CpG island is found in the promoter region of around half of all genes, and this gene arrangement has received the most attention recently [3]. In colorectal cancer, a remarkable level of abnormal methylation occurs within the CpG-rich region even though there is a global depletion of cytosine methylation in the genome. As a result, it leads to epigenetic silencing of gene expressions and subsequently, the inactivation of the relevant gene (i.e., *MLH1*) followed by mutation of tumor suppression genes encoding tumor-suppression proteins (i.e., TGFBR2 and BAX) [6]. For instance, the Hereditary nonpolyposis colon cancer (HNPCC) or Lynch syndrome is characterized by germ-line defects in mismatch repair MHL1 and *MSH2* genes due to the methylation-induced silencing phenomenon [155]. Somatic inactivation of the wild-type parental allele or more specifically, methylation-inactivated MHL1 gene is also the cause for loss of mismatch-repair function in HNPCC [156]. Therefore, the genomic pattern of HNPCC could be characterized by the combination of somatic and germ-line defects. A specific subgroup resulting from an aberrant methylation mechanism known as CpG island methylator phenotype (CIMP) is discovered in 15% of colorectal cancer cases where it is presented with MHL1 gene expressions silencing. This phenotype is categorized into 2 different subtypes: CIMP-low and CIMP-high in which the magnitude of the methylation is parallel with the clinical manifestations as moderate or aggressive respectively [157].

Cytoskeletal proteins are believed to be a potential therapeutic target as malignant cell transformation commonly displayed interactions among the mismatch-repair system, especially *MLH1* protein, due to cytoskeletal reorganization. The other cytoskeletal scaffolding proteins that are involved in such interaction include Actin gamma, Annexin A2, Cathepsin B, Desmin, and Thymosin beta 4 [158]. In CRC with *MLH1*-deficient cell lines, low levels of cytoskeletal SPTAN1 scaffolding proteins are associated with decreased cell migration whereas high levels of SPTAN1 could promote tumor progression and invasion [159,160]. Furthermore, sporadic tumors with microsatellite instability (MSI) were shown to have higher rates of promoter methylation in numerous genes, including CDKN2A, which encodes the protein INK4A, and THBS1 (thrombosponsin 1) [161]. Other investigations have included HPP1 (hyperplastic polyposis gene 1, also known as TMEFF2) and CDKN2A, which encodes ARF and other proteins, to the list of genes that are preferentially hypermethylated in sporadic MSI positive cases. [162].

### 5.2. Base Excision Repair Defects

From prokaryotic to eukaryotic cells, base excision repair has been employed to repair the high volume of endogenous DNA damage that occurs as part of the normal physiology process. It is also necessary for normal mammalian development, and its absence has been linked to neurological diseases and cancer. [163]. MutY homolog base excision repair gene (MUTYH) which encodes its MYH protein functions to excise the 8-oxoguanine product from the DNA. The product excised is due to the oxidative damage to Guanine base in the DNA strain [164]. The germ-line inactivation of MYH base-excision gene can result in the development of colorectal cancer. The risk of polyposis phenotype can reach as high as 100% in people by the age of 60 years old, who carry two inactive germline MHY alleles. Genetic testing has proven two common mutations, G382D and Y165C, that are account for 85% of cases of MYH-associated polyposis [6].

Virtual screening was used to identify cytotoxic compounds that would bind to *MSH2*/*MSH6* while the protein is in the death-signaling conformation, causing apoptosis. A DNA-*Escherichia coli* MutS “as a MSH homolog” complex modified to incorporate the cisplatin adduct cross-linking DNA and performed molecular simulation for the complex [165]. The generated ensemble of conformations was docked with a small library of commercially available drugs to determine which compounds had the highest binding affinities. It was discovered that the *E. coli* MutS-DNA complex in vitro on *MSH2*/*MSH6* may really employ a selectively binding ligand to choose the proteins’ death-signaling conformation. This study revealed the predictive capacity of in silico molecular dynamics and virtual screening for drug selection. Based on the previous work, the dynamics of MutSα-DNA complexes were studied in order to better understand the physiological response to DNA damage signaling by mismatch-repair proteins. Negureanu et al. [166] used 50 ns molecular dynamic simulations to study correlated movements in response to MutSα binding of mismatched and platinum cross-linked DNA fragments. Firstly, the protein dynamics in response to mismatched and damaged DNA recognition show that MutS signals their recognition via distinct pathways, giving support for the molecular basis of mismatch repair-dependent death. Secondly, the *MSH2* subunit is implicated in signaling both mismatched and damaged DNA recognition; localized and collective movements within the protein enable identifying locations on the *MSH2* surface that may be relevant in recruiting proteins responsible for downstream actions. This verifies *MSH2*’s involvement in signaling DNA damage-induced apoptosis and implies that deficiencies in mismatch repair alone are sufficient to cause carcinogenesis, lending credence to the experimental data that mismatch repair-damage response function might protect against tumor initiation. Identifying these specific communication locations might have significance for the treatment of malignancies that are not mismatch repair–deficient but are unable to function adequately for mismatch repair–dependent responses following DNA damage, such as cisplatin resistance.

## 6. Conclusions

The diverse yet intertwined CRC molecular pathways were reviewed, focusing mainly on the ligand–target based interactions. Furthermore, the importance of in silico studies for the genes that are having a pivotal role in changing the course of the disease was presented. After such studies, it has been found that some had an important impact on the de novo synthesis or repurposing of known commercial drugs to be used as anticancer agents. Moreover, computer-aided drug discovery facilitated the identification of lead compounds for targets that have only a partial or no crystal structure yet identified. When compared to the experimental results, in-silico techniques such as docking, pharmacophoric, shape similarity screening, and molecular dynamics were found to be significantly correlated with wet laboratory results, and this was illustrated in the examples cited in the tables above. Of note, the advances that are being made in virtual drug discovery models and algorithms are time, effort, and cost-saving in discovering new selective inhibitors for allosteric cancer targets and complicated pathways.

## Figures and Tables

**Table 1:** In silico screening studies that tackle tumor suppressor genes with a library of compounds used and the summaries of those findings.

Screening Type	Ligands	Receptor/PDB ID	Summaries	Ref.
A set of docking methods followed by molecular dynamic simulations	ZINC13, NCI, and Maybridge databases	APC-Asef/3NMZ MAI peptides/PDB: 5IZA, 5IZ6, 5B6G, 5IZ9, and 5IZ8	The main target was to prevent APC-Asef interaction that spreads CRC to the entire colon. The induced fit was performed on compounds with a variety of chemical scaffolds and direct interaction with Arg549 and other active site residues. Because of the strong interactions with Arg549, visible conformational changes occur, allowing for proper positioning inside the peptide binding region. The top hit inside the APC-Asef binding region was subjected to specific MD simulations, which revealed substantial interactions necessary for biochemical recognition in a dynamic microenvironment.	[59]
Structure-based virtual screening by rigid and flexible docking followed by in vitro assays	13.3 million drug-like and 89.4 natural product compounds	TNKS-1/2RF5 TNKS-2/3KR8	This study targets the WNT/β-catenin pathway by developing inhibitors against tankyrase 1/2. Out of 11 structurally representative top hits, one compound was selected for experimental analysis	[60]
Structure-based virtual screening followed by biological assays	500,000 structurally diverse compounds	Homology modeling of the closely related Smoothened receptor (PDB ID: 4JVK)	The study’s aim was to screen ligands targeting the transmembrane domain of frizzled protein-7 Fzd7. Fzd7 inhibitors have been identified in six small molecule drugs. With IC50 values in the sub-micromolar range, the strongest hit, SRI37892, effectively suppressed Wnt/Fzd7 signaling.	[61]
High-throughput, and ligand docking-based virtual screening	20,000 natural products	Human Telomeric DNA/1KF1	Using the X-ray crystal structure of the intramolecular human telomeric G-quadruplex DNA, a model of the intramolecular G-quadruplex loop isomer of NHE III1 was created. The aim of this study is to stabilize the c-myc G-quadruplex. The naphthopyrone fonsecin B was found the top candidate.	[62]
Binding site identification, drug design, and large-scale virtual screening	4.7 million compounds from ZINC12 drug-like subset	Myc-Max recognizing DNA/1NKP	A binding site on the structurally organized Myc-Max complex’s DNA-binding domain was discovered. Computer-aided drug design was employed to identify a small molecule that can inhibit Myc-Max functionality. In vitro analysis found a chemically different scaffold inhibitor than the previously identified Myc inhibitor.	[63]
A comprehensive molecular docking and bioinformatics analysis followed by in vitro assays	NSC765600 and NSC765691, derived from diflunisal and fostamatinib respectively	CCND1/6P8G CDK4/4O9W PLK1/2W9F and CD44/1UUH	CCND1/CDK4/PLK1/CD44 were identified as target genes for NSC765600 and NSC765691 compounds by target prediction tools. In numerous cancer types, the mRNA levels of CCND1/CDK4/PLK1/CD44 were greater in tumor tissues than in normal tissues. Protein-protein interaction networks among those genes have been shown after taking into account the gene neighborhood, gene fusion, gene co-occurrence, and the coexpression of CDK4 with CCND1, CD44, and PLK1, and CCND1 with PLK1 have been illustrated. The antiproliferative and cytotoxic effects of the 2 compounds against a panel of NCI-60 cancer cell lines have been illustrated.	[64]
A comprehensive molecular docking and bioinformatics analysis followed by in vitro assays	Sulfasalazine	KRAS/6BP1, MMP7/2Y6C and CD44/1UUH	The molecular docking revealed a unique interaction between sulfasalazine and KRAS, MMP&, and CD44. Bioinformatic analysis identified overexpression of those oncogenes in CRC cells. The synergistic effects of the sulfasalazine and cisplatin were successful in reducing cell viability, colony, and sphere formation in CRC cell lines. Sulfasalazine therapy reduced KRAS/MMP7/CD44 expression in CRC cell lines in a dose-dependent fashion.	[65]
Molecular docking and virtual screening followed by in vitro and in vivo assays	13,000 diverse small molecules from the ZINC database	ND	68 compounds were identified from the screening to interact with the binding site of α5β1-integrin. By inhibiting the urokinase receptor/integrins interaction, 2-(Pyridin-2-ylamino)-quinolin-8-ol and 2,2′-(methylimino)di (8-quinolinol) suppressed ERK activation. In vivo, these two drugs suppressed ERK activation, tumor development, and metastasis in a model head and neck cancer.	[66]
Protein binding pocket prediction and structure-based virtual screening	5000 chemical compounds collected from ZINC were chosen based on structural similarity indices to the four ligand probes	GSK3β/3DU8	A protein binding pocket screening was done on an X-ray model of human GSK3 beta using the geometric analysis via the Voronoi tessellation algorithm. Pocket geometry is the most important factor in ligand binding. Using molecular docking to find probable binding sites yielded comparable results to protein pocket prediction.	[67]
Computational drug-repositioning approach for identifying novel anti-cancer agents	973,296 chemical–gene interactions from Comparative Toxicogenomics Database including 7570 chemicals/drugs and 20,116 genes	ND	DrugPredict platform was employed to repurpose chemicals and drugs for endothelial ovarian cancer. Indomethacin decreases cell viability and promotes apoptosis in patients with primary high grade severe cancer-derived cell lines. Because it inhibits β-catenin and represses multiple Wnt signaling targets, such as Lgr5, TCF7, and Axin2, it proved effective against platinum-resistant ovarian cancer cells.	[68]
Virtual screening by molecular docking followed by in vitro assays.	1990 small molecules from the National Cancer Institute database	β-catenin/Tcf4 complex (PDB/1JPW chain A)	Site A hotspot on beta-catenin was chosen as a virtual screening pharmacophore. The top-ranked molecule has effectively reduced the β-catenin/Tcf4 driven activity in the CRC cell line. It prevents β-catenin from directly binding to Tcf4 and suppresses the expression and activity of Wnt/β-catenin target genes and gene products.	[69]
New binding pockets detection, structure- and ligand-based virtual screening, molecular dynamics simulations, and binding free energy calculations	1880 structures from diversity Set II were obtained from the ZINC database. 50 structures from the above were selected for similarity screening from the ZINC15 database	Domain 1 and 2 of LRP6/4DG6, domains 3 and 4 of LRP6/4A0P	After applying Lipinski’s rule of five and flexible molecular docking, ten candidate compounds were found, five of which were for each binding pocket. It has been concluded that ZINC03954520, ZINC01729523, ZINC03898665, ZINC13152226, ZINC26730911, and ZINC01069082 are possibly appropriate compounds for inhibiting LRP6 using RMSD, RMSF, the radius of gyration, and MMPBSA binding free energy calculations.	[70]
Ensemble docking-based virtual screening	3520 natural products	Tp53/1TSR	Natural products were screened to identify a ligand that stabilizes the function of the wild type p53 by targeting its Loop1/Sheet3 pocket. Due to the flexibility of Loop1, ensemble docking for 7 conformations was performed. Compound torilin not only enhanced p53 activity but also p21 protein production, which is downstream of p53.	[71]
The Nanoluc/YFP-based bioluminescence resonance energy transfer (BRET) test was combined with structure-based virtual screening and followed by	Commercially available protein-protein interaction small molecules from ChemDiv	Bcl-xL/2YXJ	The purpose of this study is to find inhibitors of Bax/Bcl-xL and Bak/Bcl-xL interactions. Based on BRET techniques, a screening platform for Bak/Bcl-xL and Bax/Bcl-xL interactions were developed and identified inhibitors of both interactions. ABT-737, an inhibitor for Bcl-xL, was employed as a positive control drug to identify more inhibitors. 50 Compounds were selected via virtual screening that targeted the ABT-737 binding site and only BIP-A1001 and BIP-A2001 showed dose-response inhibition for the Bax and Bcl-xL interactions within low micromolar concentration	[72]
Pharmacophore- and structure-based virtual screening	582,474 compounds from TimTec Compound Libraries	MDM2/3JZK	Based on a conventional Mdm2 inhibitor, a set of pharmacophoric characteristics was developed and utilized to screen a ligand library, and the potential inhibitors were docked into the receptor to check their potential to stop MDM2-p53 interaction. Triazolopyrimidine was among top 5 compounds that bind to the MDM2 active site.	[73]
Pharmacophore virtual screening and molecular dynamic simulations.	National Cancer Institute and ZINC Libraries	Caspase-9/1JXQ	Due to a substantial missing section of the crystallographic structure, the caspase-9 structure was refined. Four structures were employed with PDB IDs of 4DGE, 4DGA, 2PBj, and 1Z9H to build the missing part. For evaluating the ligands’ forms of interaction in the protein binding pocket, a pharmacophore model approach was applied. The compound selected from pharmacophore screening and rigid docking was further checked for binding pose stability through MDS with stable hydrogen bonds.	[74]
Structure- and ligand-based 3D pharmacophore models followed by in vitro assays	50,000 compounds from Maybridge database	Caspase-3/1pau	Using 25 various compounds, a ligand-based pharmacophore model was generated. Further docking experiments on known inhibitors revealed that the amino acids Arg207, Ser209, and Trp214 found in the active region of caspase-3 are critical for ligand binding. From this study, methyl piperazine was identified as a non-peptide inhibitor against Caspase-3.	[75]
Homology modeling for predicting target protein sequence and virtual screening for finding inhibitors	Mcule database was used for small molecule virtual screening	TNFRSF10B/2ZB9, 3NKE, 3NKD	TNFRSF10B best model was built by using 2ZB9 template and assessed by 3 different software with high scores. An evolutionary tool was employed to construct a neighbor-joining tree of the target gene based on TNFRSF10A, TNFRSF10D and TNFRSF10B genes. Virtual screening revealed 4 lead compounds with inhibitory activities against the mutated TNFRSF10B activity. To investigate the highly interacting proteins of the target protein, a functional partner network of the TNFRSF10B protein was created. TNFSF10 was utilized as a ligand-protein in protein-protein docking because it had the greatest interaction.	[76]
Virtual screening (pharmacophoric molecular identification), molecular docking, followed by molecular dynamics and experimental assays	8 million compounds from a clean and drug-like subset of the ZINC database, and 260,071 compounds from the NCI-2003 library	The crystal structure of TGF-b3 in complex with the extracellular domain of TßRII/1KTZ	The main purpose of this study was to discover drugs that antagonize TGF-b signaling by protein-protein competitively inhibiting TGF-b binding to TßRII. Two compounds were found with a quite good binding affinity (26 and 18 µM). Three compounds were found to bind to SS1 on TßRII over the duration of the simulations, according to molecular dynamics trajectories. The 3 compounds share the chemical property of being aromatic and fairly flat	[77]
Shape-based virtual screening followed by experimental work and X-ray crystallization study for TGFb-1 inhibitor	200,000 Compounds in the multi-conformational Catalyst database	The pharmacophoric query was constructed using SB203580′s conformation as shown in the X-ray combination with p38 (PDB: 1a9u).	The pharmacophore features were chosen based on a derived alignment of p38-SB203580 (a triarylimidazole) with TβRI’s ATP site. 87 compounds were identified satisfying both the shape constraint and pharmacophore features. With IC50 of 60 nM, HTS466284 was found to be a strong, non-toxic inhibitor of TßRI in vitro and in cell culture. The aromatic contacts of the HTS466284 indicated by the shape question are satisfied by the quinoline, pyrazole, and pyridyl rings.	[78]
*De novo* synthesis of caspase-6 inhibitors using neural network, and molecular docking-based ligand screening	2.4 million molecules were retrieved from PubMed to train the RNN model	caspase-6/3OD5	For *de novo* molecular design of caspase-6 inhibitors, a gated recurrent unit (GRU)-based RNN network was merged with transfer learning and classical machine learning. A prediction model was trained on known caspase-6 inhibitors and decoys. The 6927 synthesized inhibitors that were developed share the same chemical space as the known caspase-6 inhibitors. The synthesized inhibitors are predicted to have comparable binding mechanisms to the known 577 caspase-6 inhibitors.	[79]

**Table 2:** An overview for some Vascular Endothelial Growth Factor Receptor-2 inhibitors, their PDB-ID, resolution, and their effects on other receptor kinase targets.

VEGFR2 Inhibitor	PDB ID	Resolution	Comments	Inhibitor Type/other RTKs Inhibition
Sorafenib	4ASD	2.03 Å	Mutated	Type IIA, also inhibits VEGFR2/3, BRaf, CRaf, mutated BRaf, Kit, Flt3, RET and PDGFRB
Axitinib	4AG8	1.95 Å	Mutated	Type IIA, also inhibits VEGFR2/3, PDGFRB
Sunitinib	4AGD	2.81 Å	Mutated	Type I, also inhibits PDGFRB/alpha, VEGFR2/3, Kit, Flt3, CSF-1R, and RET
Pazopanib	3CJG	2.25 Å	Not mutated	Type I, also inhibits PDGFRB/alpha, VEGFR2/3, FGFR1/3, Kit, Lck, Fms, Itk.
Lenvatinib	3WZD	1.57 Å	Mutated	Type I1/2A, also inhibits PDGFR, VEGFR2/3, FGFR, Kit, RET
PF-00337210	2XIR	1.50 Å	Mutated	Type II inhibitor
CHEMBL272198	3B8R	2.70 Å	Mutated	Type I, also inhibits Aurora B, ABL1, c-MET, Tie2, Lck, Lyn
CHEMBL194911	1YWN	1.71 Å	Mutated	Tie-2 and VEGFR2 dual inhibitors
2-Anilino-5-aryloxazole	1Y6A	2.10 Å	Not mutated	
LENVATINIB	3WZD	1.57 Å	Mutated	Also inhibits VEGFR2/3, PDGFR, FGFR, Kit, RET
TIVOZANIB	4ASE	1.83 Å	Mutated	Pan-inhibitor of VEGF receptors
MOTESANIB	3EFL	2.20 Å	Mutated	Inhibitor of VEGF, PDGF, and Kit receptors

**Table 3:** Summaries of high throughput virtual screening that aim at finding hits against vascular endothelial growth factor receptor-2.

Screening Method	Database Size	Summaries	Ref.
High throughput virtual screening for EGFR inhibitors	400,000 compound library of tyrosine kinase inhibitors from ChemBioBase	Indenopyrazole framework was reported as cyclin-dependent kinase inhibitor. The framework was discovered to be one of the most prevalent structures among the top 100 scoring compounds, prompting the development of a series of indenopyrazoles. Interestingly, some of the synthesized compounds suppressed VEGFR-2 tyrosine kinase at 1 micromolar.	[97]
Molecular docking, multicomplex pharmacophore and fingerprint-based 2D similarity in an individual and a combined manner.	409 actives and 24,680 decoys	In a retrospective comparison, the three combined approaches outperformed 43 of 45 previously published articles. The results showed that the 2D fingerprint ECFP 4 outperformed the multicomplex pharmacophore Glide SP. In self- and cross-docking studies, Glide SP docking with PDB ID: 3EWH was shown to be the best choice for molecular docking-based screening.	[98]
Molecular flexible docking followed by virtual screening, pharmacophore and ligand energy inspection	284 compounds from the PubChem database were found with the highest similarity with the best active compound.	Among 23 inhibitors, compound CHEMBL346631 (Pubchem CID: 9936664) was identified as the highest efficient ligand interaction with VEGFR2. The greatest affinity against Renal Cell Carcinoma was found in the dicarboxamide (SCHEMBL469307) from the PubChem database. The original inhibitor chemical is more stable in the receptor protein than the virtually screened one.	[99]
Virtual screening followed by molecular dynamics and binding free energy decomposition calculations	30,792 natural derivatives from the ZINC 15 database	Three 1-azabicyclo [2.2.2] octane-3-carboxamide derivatives with excellent affinity were discovered using the VEGFR2 inhibitor as a reference to uncover more inhibitors from natural resources. These potential molecules might be VEGFR-2 inhibitors, according to the RMSD study of each VEGFR-2–inhibitor combination, in addition, they showed low binding free energy and decomposition energy for each VEGFR-2–inhibitor interaction.	[100]
Virtual screening by using homology models, pharmacophore modeling and in vitro studies	46 derivatives of 2-anilino-5-phenyloxazoles	As VEGFR2 inhibitors, two 2-anilino-5-phenyloxazole derivatives were shown to be effective. Because the crystal structure of VEGFR2 was not available at the time of this work, homology models were employed instead. At the ATP-binding region, the compounds shared a pharmacophore and established hydrogen bonds with the backbone’s Cys919. The activation loop was disordered between residues 1046 and 1065 in both crystal structures, indicating that residues beyond this region were not directly contributing to the binding affinity.	[101]
Structure-based pharmacophore models followed by virtual screening of several commercial databases.	Key Organics (48,768), Maybridge (94,448), Otava (69,700), Life Chemicals (248,445), Asinex (358,126)	Following pharmacophore modeling, 16,000 and 19,000 compounds were identified as type I and type II inhibitors respectively. A total of 100 compounds were taken to biological testing after the flexible docking. Three compounds with excellent binding and drug-like characteristics were discovered. The 3-membered ring of the triazinoindole derivative (IC50 = 1.6 micromolar) establishes two standard hydrogen bonds with the backbone NH and the carbonyl oxygen of Cys917 in the kinase motif (type II).	[102]
De novo structure-based identification methods followed by in vitro assays	A range of pyrazole-based compounds was designed to be employed.	Using a structure-based de novo design, the researchers discovered a new VEGFR2 inhibitor scaffold. As a multi-tyrosine kinase inhibitor, this resulted in the development of a pyrazole-based molecule (JK-P3) that targets VEGFR2 kinase activity and angiogenesis while also inhibiting FGFR kinases in vitro.	[103]

**Table 4:** The characteristics of virtual screening, protein kinases, and the resulting compounds of the screening.

Screening Type	Ligands	Receptor/PDB ID	Findings	Ref.
Structure-based screening	Curcumin, litreol, triterpene	EGFR/3POZ	The predicted pharmacological features of curcumin were found to be better than litreol and triterpene.	[126]
Pharmacophore and docking screening for Korean *P. ginseng* active compounds	128 ginsenosides	EGFR/1M17	Molecular docking studies identified 14 hit molecules based on scoring function and suitable binding orientation with critical active site amino acids.	[127]
The combination of docking and molecular dynamics simulation had been carried out to design new quinazoline derivatives compounds	Erlotinib, Afatinib, and WZ4002 were optimized into A1, B1, and C1 lead compounds.	EGFR/1M17	Molecular docking was successful in designing new potential compounds using the pharmacophore model of lead compounds. The interaction between lead compounds and the receptor was evaluated by MMGBSA. A1 is a potential compound as an EGFR inhibitor.	[128]
Structure-based virtual screening	615,462 compounds were obtained from the ZINC database	EGFR/1M17	Six compounds displayed good effects when compared with erlotinib at 30 μM. At 2 μM, one compound showed inhibiting effects close to those from erlotinib.	[129]
Structure-based virtual screening for non-small cell lung cancer (NSCLC)	93 million compounds obtained from the PubChem database	AKT/3AOX	The virtual screening showed that (PubChem CID123449015) is more efficient to be a better prospective candidate for NSCLC treatment having better pharmacological profile than the pre-established compound PubChem CID71721648 with low toxicity and cytotoxicity	[130]
Structure-based screening for repurposing of an antifungal drug against gastrointestinal stromal tumors	A docking with 36 antifungal drugs and 5 antineoplastic drugs.	PDGFRA/5K5X	Itraconazole was predicted as a better PDGFRA inhibitor among all the computationally tested drugs. The binding affinity of Imatinib was close to that of Itraconazole.	[131]
Structure-based virtual screening toward the experimental DNA G-quadruplex (G4s) structures of *c-myc* and *c-Kit*	693,000 commercial compounds obtained from Asinex	*c-myc*/1XAV and 2L7V *c-Kit*/4WO2, 4WO3 and 2O3M	Ensemble docking simulations resulted in 442 for *c-myc* and 634 molecules for *c-Kit* G4s. The 76 shared hits in complex with both receptors investigated for their thermodynamic behavior. Three N-(4-piperidinylmethyl)amine derivatives effectively stabilized both G-quadruplex oncogene promoter structures	[132]
Machine learning-based virtual screening with multiple PI3Kγ protein structures.	87 crystallographic structures of PI3Kγ-inhibitor complexes	PI3Kγ/4wwo, 5g2n, 3r7q, 3ml8, 2a5u, 4flh, 4fjy, 4ps7, 2v4l, 3dbs	The developed NBC model integrating ten PI3Kγ proteins showed a satisfactory prediction power against PI3Kγ inhibitors. JN-KI3 ligand exhibits the most potent selective inhibitory bioactivity. The results of molecular docking, MD simulation, and free energy calculations reveal that JN-KI3 contains the highest binding free energy against PI3Kγ than Class IA isoforms.	[133]
A support vector machine as a virtual screening tool for searching Abl inhibitors from large compound libraries	13 and a half Million PubChem, 168K MDDR, and 6 638 MDDR molecules	Similarity screening with known Abl inhibitors	The model shows substantial capability in identifying Abl inhibitors at substantially lower false-hit rate. 29 072 inhibitors (0.21%) of 13.5 M PubChem lib. 659 inhibitors (0.39%) of 168K MDDR lib. 330 (5.0%) of 6 638 MDDR lib.	[134]
A structure- and ligand-based virtual screening were involved to investigate ligands targeting the allosteric site of Abl kinase	1424 compounds from DrugBank database v3.0	Abl/3K5V	A series of in silico techniques like virtual screening, molecular dynamics, and steered molecular dynamic simulations were employed. Gefitinib was identified as an inhibitor for over-expressing Bcr-Abl protein in the K562 CML cell line. It was found that the combination of imatinib and gefitinib produced a synergistic antiproliferative effect in such a cell line.	[135]
High Throughput Virtual Screening, Standard Precision, and Extra Precision docking, followed by molecular dynamic simulations.	Natural product libraries of ZINC database and Drug bank database	Abl1/3QRJ	Comparative docking analysis was also carried out on the active site of the ABL tyrosine kinase receptor with a reported reference inhibitor. The purpose was to identify inhibitors for mutated BCR-ABL protein. Six inhibitors were further validated and analyzed through pharmacokinetics properties and a series of ADMET parameters by in-silico methods	[136]
Structure-based pharmacophore modeling, virtual screening, and molecular docking simulations	200,000 commercially compounds	14-3-3σ isoform/1YWT	The purpose was to design a small molecule able to inhibit protein–protein interactions between 14-3-3 and c-Abl. BV02 which was designed by in silico process is a terephthalic acid derivative and was found as an anti-proliferative on human leukemia cells either sensitive or resistant to Imatinib due to the T315I mutation. It also mediates c-Abl release from 14-3-3 protein.	[137]
High throughout virtual screening for calculating the binding score, hydrogen bonds, and hydrophobic complementarity, and free energy of binding.	300,000 molecules from the SPECS subset from the Zinc. The database was filtered down to 90,000 for compounds with a logS value of greater than—4 for better solubility	BRaf/2FB8	Identification of a series of purine-2,6-dione analogs that are selective for BRaf. The best lead compound inhibits the kinase activity of BRAF with an IC50 value of 1.7 μM and high selectivity compared to other protein and lipid kinases.	[138]
A virtual docking screening along with pharmacokinetics and drug-likeness predictions to find V600E-BRAF inhibitors.	Eleven derivatives of 4-(quinolin-2-yl) pyrimidin-2-amine.	V600E-BRAF/3OG7	Two derivatives of 4-(quinolin-2-yl) pyrimidin-2-amine were found to have binding patterns similar to that of the vemurafenib the drug used against V600E-BRAF malignancies. It is also indicated that the compounds had more favorable ligand-protein interaction energy than vemurafenib at the binding site of V600E-BRAF	[139]
Computer-aided drug discovery including pharmacophore modeling, molecular docking, and molecular dynamic simulations for finding KRAS G12D potential inhibitors	More than 214,000 compounds from InterBioScreen and ZINC databases	KRAS G12D/6GJ8	Firstly, a common pharmacophoric feature model was generated to extract the important criteria for KRAS inhibition. Ligands from databases were mapped on the model and mapped compounds were finally subjected to molecular docking and dynamic simulations. Four potential inhibitors displaying favorable stability with KRAS G12D were obtained, and only 2 of them showed better binding free energies.	[140]
Fragment-based drug design was conducted to inhibit KRAS-PDEδ protein–protein interactions	Quinazolinone and f benzimidazole fragments that are attached with PDE gamma	PDEδ/5×73 PDEδ/4JV6	A combination of the two fragments produced novel quinazolinone-imidazole KRAS-PDEδ inhibitors. The experimental results approved the high binding affinity and antitumor activity of this compound.	[141]
Structure-based screening for molecular binding interactions binding affinities	49 Artemisinin derivatives	HDAC2/3C0Z ERK1/4QTB ERK2/5NGU	It has been found that artemisinin dimer and artemisinin dimer hemisuccinate are promising anticancer drug agents, with better therapeutic efficacy than the standard inhibitors; ulixertinib and apicidin for the treatment of cancer via inhibition of ERK1, ERK2 and HDAC7.	[142]
Scaffold hopping, followed by fragment-based drug discovery and molecular dynamics simulations	The ERK2 inhibitor Ulixertinib was used for scaffold hopping.	ERK2/6GDQ	Initial hits retained from scaffold hopping usually are not enough for finding potential hits. FBDD can be employed for improving the binding potential of the hopped hits. The identified ligands showed good binding affinity similar to Ulixertinib	[143]
Structure-based pharmacophore study, followed by virtual screening	200,158 compounds from the SPECS library	(MAP2K2) MEK2/3DV3	The pharmacophore model of MEK1 inhibitors was constructed and used for a large-scale virtual screening. 13 virtual hits against MEK1 were obtained from the SPECS library. Then, a small library of carbazoles was synthesized based on one hit by bioisosteric replacement with IC50 at the micromolar level of allosteric inhibition of MEK2.	[144]
Docking analysis, and pharmacophore modeling study	350 anticancer natural products.	HER2/3RCD	The hits were selected for the comparative study with the established HER2 inhibitors lapatinib and neratinib and interactions were studied. Finally, the pharmacophoric model was built. Eight natural products were obtained as hits by virtual screening and the comparative study. Results revealed that mostly anthocyanidins have the potential to target the kinase domain of HER2.	[145]
2D, 3D quantitative structure–activity relationship (QSAR) and pharmacophore studies.	725 hits World Drug Index (WDI) and 19,773 from ChemBridge.	IGF-1R/5HZN	Virtual screening of structurally diverse ligands of dual inhibitors of IGF-1R and insulin receptor. Alignment independent molecular descriptors were established for 3Dconformations. Dual potential inhibition of IGF-1R and IR was found for Tirofiban, Practolol, Edoxaban, Novobiocin	[146]
Structure-based virtual screening, molecular docking, molecular dynamics simulation and ADME prediction	A set of compounds from the NCI database in addition to naringin	PTEN/1D5R	Naringin was found to have better binding with PTEN among the 5 top-ranked compounds, docking scores and energy. The pharmacokinetic properties, Lipinski’s rule violations and binding stabilities of naringin have achieved the best results.	[147]
Structure-based virtual screening followed by biological evaluation	35,367 compounds from SPECS	AKT-1/3MVH	Two compounds were identified as AKT inhibitors with micromolar activity and high selectivity index against cancer cell lines.	[148]
bi- and three-dimensional physical-chemical filtrations followed by phenotypic assays.	5.9 million compounds from eMolecules database	mTOR/4JT5 PI3Kα/4JPS	The aminopyridine scaffold was found to target the PI3K-AKT-mTOR pathway especially the mTOR and PI3Kα proteins. This kind of drug discovery produced soluble, stable, membrane-permeable and highly selective compounds.	[149]
Pharmacophore-based virtual screening, molecular docking, and binding free energy calculations study. The structural design of cyclic peptides also included	Three databases; TOS Lab 39,988 CPP 1411 and ASINEX 31,500 compounds	PI3Kα/4KYN	compounds having indole and benzothiazole moieties can act as potent inhibitors against PI3Kα. Linear and cyclic compounds were found to be effective for PI3Kα. 1, 3, 4-oxadiazole-based cyclic peptides with tryptophan showed that cyclic peptides can act as good inhibitors against PI3Kα	[150]
Virtual inverse screening followed by biological assays	Indirubin-3′-oxime (IOX) and three derivatives of bromo-indirubin-3′oxime; 5BIO, 6BIO, and 7BIO were screened against 6000 protein binding sites	5 BIO: CDK2/1pxo 6 BIO: GSK3B/1q41 PDK1/1oky 7 BIO: RIFK/1nb9 IOX: CDK2/1pxp	The purpose is to identify kinase targets for three derivatives of indirubin; 5BIO, 6BIO, and 7BIO. 5BIO, 6BIO (EF = 16) and IOX (EF = 20) show significant enrichment of their well-known targets (CDK2, CDK5, GSK-3β) in the top 1%. This process has led to the identification of the kinase PDK1 as an unknown target of the indirubin derivative 6BIO.	[151]
Ligand-based screening, rigid and flexible receptor-based docking, molecular adynamic simulations and binding free energy calculations	688,086 compounds from ZINC 15 were reduced to 157,623 compounds after the pre-screening process.	PDK1/2BIY	The compounds were first screened by using the ligand-based method, then rigid docking, followed by flexible molecular docking using, molecular dynamics simulation and molecular mechanics/Poisson–Boltzmann surface area (MM-PBSA) binding free energy calculations. The resulted compound inhibited many other cancer cell lines, such as multiple myeloma, non-small cell lung cancer, colon cancer, CNS cancer cells, Melanoma cell, Ovarian cancer cells, Renal cancer cells, Prostate cancer, and Breast cancer cell lines.	[152]
Ensemble docking to disrupt protein–protein interactions followed by rescoring with the molecular mechanics Poisson–Boltzmann surface area (MM/PBSA)	84,589 compounds were studied by Xiao et al. [153]	FGF23/2P39 In addition to the homology of three crystal structures, two of FGF19/1PWA and 2P23 one of FGF12/1Q1U FGFR1/1FQ9	The target selected has only a partial crystal structure and no a priori knowledge of small-molecule binding sites. Two putative binding sites for drug-like antagonist molecules binding to the hormone FGF23 were identified using a multicenter ensemble docking technique. The use of MM/PBSA rescoring to further enhance the MED results demonstrates the value of going from lower-resolution approaches to higher-resolution methods for refining a predicted binding mode. This study also reveals how the steric crowding of pockets by side-chain conformers might affect docking outcomes. Authors hypothesized that the protein–protein interface is being drugged and not a distal pocket that would indicate allosteric signaling	[154]
